# Hemorrhagic Bullous Henoch-Schönlein Purpura: Case Report and Review of the Literature

**DOI:** 10.3389/fped.2018.00413

**Published:** 2019-01-22

**Authors:** Matthias Nothhaft, Joerg Klepper, Hermann Kneitz, Thomas Meyer, Henning Hamm, Henner Morbach

**Affiliations:** ^1^Department of Pediatrics, University Hospital Würzburg, Würzburg, Germany; ^2^Department of Pediatrics, Klinikum Aschaffenburg-Alzenau, Aschaffenburg, Germany; ^3^Department of Dermatology, Venereology and Allergology, University Hospital Würzburg, Würzburg, Germany; ^4^Department of Pediatric Surgery, Pediatric Traumatology and Pediatric Urology, University Hospital Würzburg, Würzburg, Germany

**Keywords:** henoch-schönlein purpura, vasculitis, hemorrhagic, bullae, children

## Abstract

Henoch-Schönlein Purpura (HSP) or IgA vasculitis is the most common systemic vasculitis of childhood and may affect skin, joints, gastrointestinal tract, and kidneys. Skin manifestations of HSP are characteristic and include a non-thrombocytopenic palpable purpura of the lower extremities and buttocks. Rarely, HSP may initially present as or evolve into hemorrhagic vesicles and bullae. We present an otherwise healthy 5-year-old boy with an acute papulovesicular rash of both legs and intermittent abdominal pain. After a few days the skin lesions rapidly evolved into palpable purpura and hemorrhagic bullous lesions of variable size and severe hemorrhagic HSP was suspected. A histological examination of a skin biopsy showed signs of a small vessel leukocytoclastic vasculitis limited to the upper dermis and direct immunofluorescence analysis revealed IgA deposits in vessel walls, compatible with HSP. To further characterize the clinical picture and treatment options of bullous HSP we performed an extensive literature research and identified 41 additional pediatric patients with bullous HSP. Two thirds of the reported patients were treated with systemic corticosteroids, however, up to 25% of the reported patients developed skin sequelae such as hyperpigmentation and/or scarring. The early use of systemic corticosteroids has been discussed controversially and suggested in some case series to be beneficial by reducing the extent of lesions and minimizing sequelae of disease. Our patient was treated with systemic corticosteroids tapered over 5 weeks. Fading of inflammation resulted in healing of most erosions, however, a deep necrosis developing from a large blister at the dorsum of the right foot persisted so that autologous skin transplantation was performed. Re-examination 11 months after disease onset showed complete clinical remission with re-epithelialization but also scarring of some affected areas.

## Introduction

Henoch-Schönlein Purpura (HSP) or IgA vasculitis is the most common systemic vasculitis of childhood and may affect skin, joints, gastrointestinal tract, and kidneys (1). Skin manifestations of HSP are characteristic and include a non-thrombocytopenic palpable purpura of the lower extremities and buttocks. Rarely, HSP may initially present as or evolve into hemorrhagic vesicles and bullae resulting in cutaneous necrosis (2). We present the case of a HSP patient with hemorrhagic-bullous skin lesions and by summarizing additional reported cases aimed at characterizing the clinical picture of bullous HSP and discussing treatment options.

## Case Report

**Figure 1:**
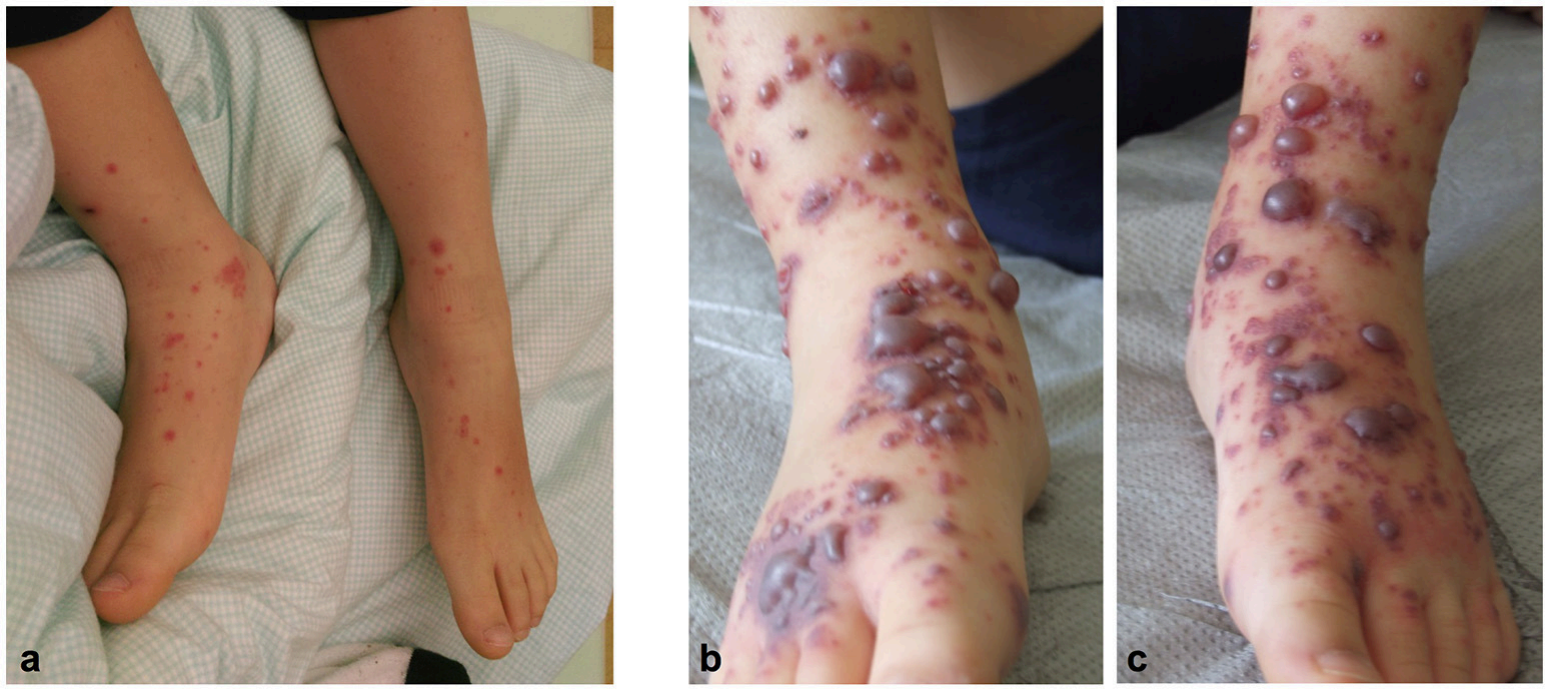
An acute papulovesicular rash of both legs **(a)** rapidly evolved into palpable purpura and hemorrhagic-bullous lesions of variable size ranging from 5 to 40 mm **(b,c)**.

An otherwise healthy 5-year-old boy presented with an acute papulovesicular rash of both legs (Figure 1a) and intermittent abdominal pain. The patient did not have fever. Differential blood count (white blood cell count of 12,900/μl with 60% granulocytes and 29% lymphocytes), C-reactive protein (0.45 mg/dl), erythrocyte sedimentation rate (20 mm/h) and global blood clotting tests (INR 0.98, PTT 31.5 s) were normal. Serum IgA (147 mg/dl) and IgM (66 mg/dl) levels were within age matched reference ranges whereas IgG levels were slightly decreased (557 mg/dl, reference range 640-1420). No hematuria or fecal occult blood could be detected. Abdominal ultrasound could exclude intussusception but revealed thickened bowel wall at the ileocecal junction. The abdominal symptoms resolved spontaneously within 2 days but arthralgia appeared thereafter.

**Figure 2:**
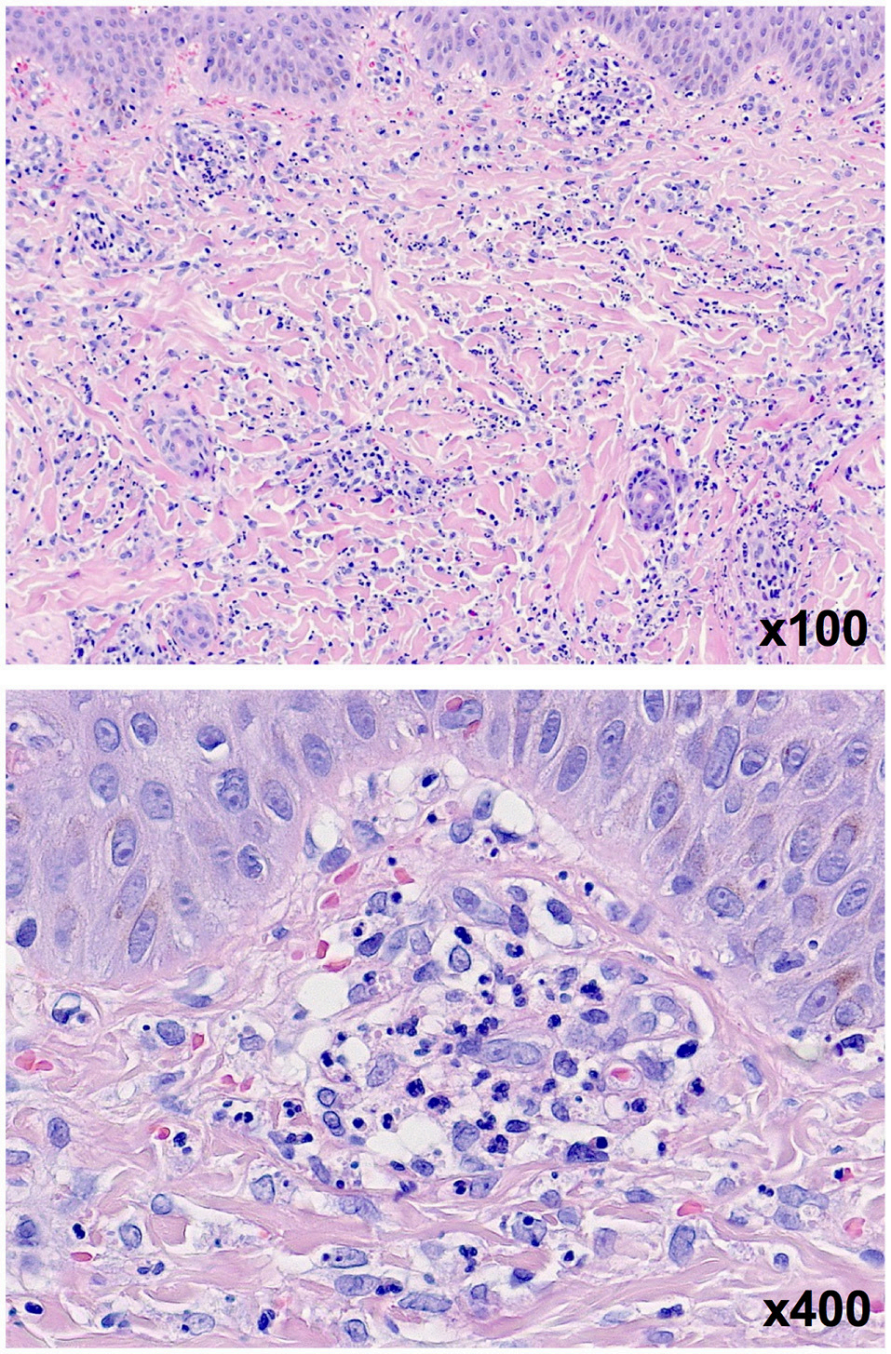
Skin biopsy of one lesion in H&E staining revealing signs of a small vessel leukocytoclastic vasculitis limited to the upper dermis.

Nine days after the onset of disease the skin lesions at the arms, legs, feet and ankles rapidly evolved into palpable purpura and hemorrhagic-bullous lesions of variable size ranging from 5 to 40 mm (Figures 1b,c). Some of the blisters spontaneously ruptured and disclosed hemorrhagic fluid which remained sterile in the microbiological work-up. The patient was given cefuroxime as antibiotic prophylaxis. Severe hemorrhagic-bullous HSP was suspected but differential diagnoses included septicemia/septic emboli and autoimmune blistering disease. Absence of fever and leukocytosis and sterile blood cultures argued against an infectious etiology. Neither circulating antibodies directed against structural proteins of the basement-membrane zone nor ANAs or ANCAs could be detected in the patient‘s serum. C4 levels were in the normal range while C3c levels were slightly elevated (152 mg/dl, reference range 80-120). A skin biopsy was performed and histological examination showed signs of a small vessel leukocytoclastic vasculitis limited to the upper dermis (Figure 2), and direct immunofluorescence analysis revealed IgA and C3 deposits in vessel walls, compatible with HSP.

**Figure 3:**
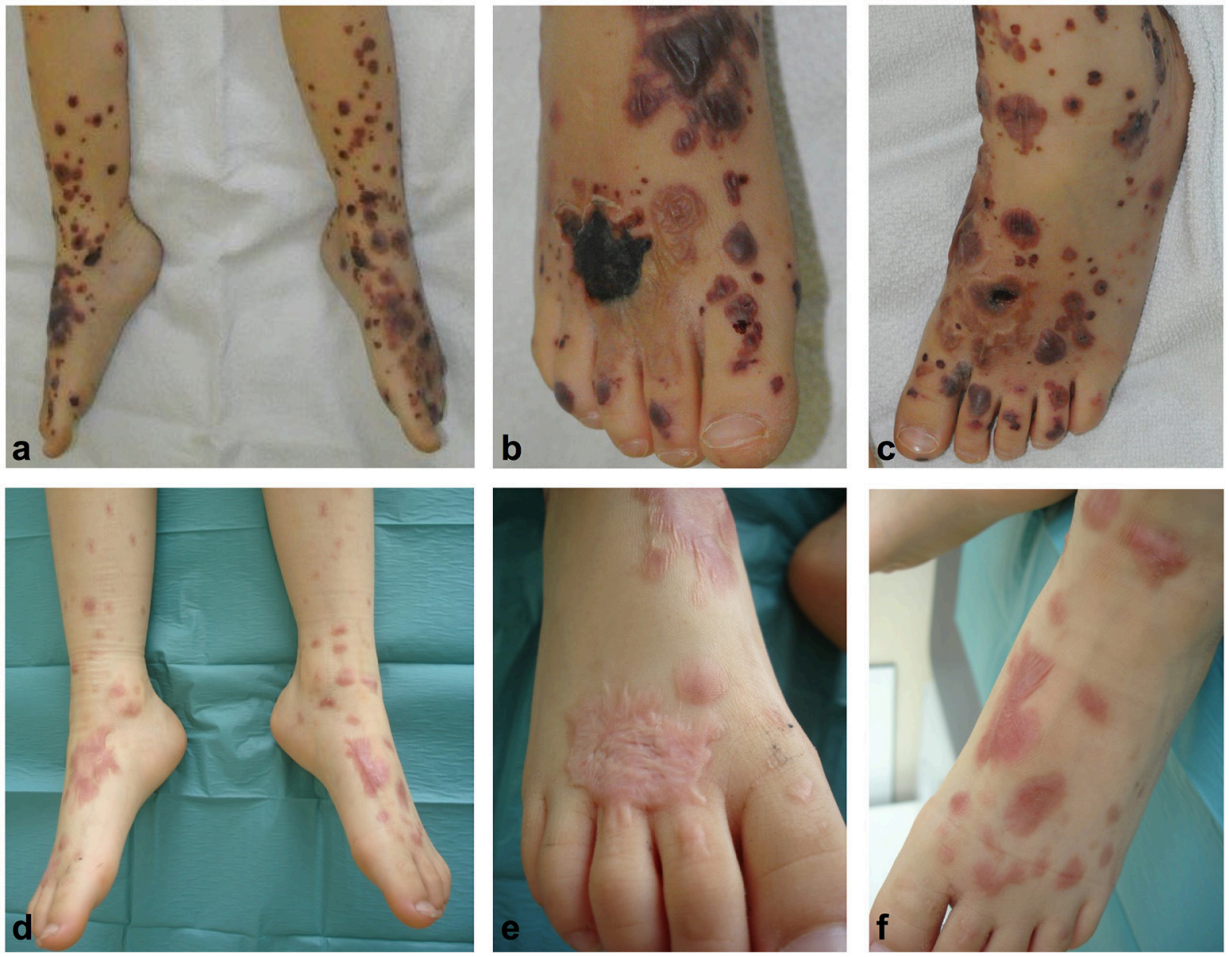
Hemorrhagic bullae developed on both feet an lower legs **(a–c)**. A deep necrosis resulting from a large blister at the dorsum of the right feet evolved **(a,b)** neccessitating autologous skin transplantation. Re-examination 11 months after disease onset showed complete clinical remission of disease with re-epithelialization of affected areas **(d–f)**.

The patient was treated with oral corticosteroids (prednisolone 1 mg/kg/day) for 7 days, then subsequently tapered over 39 additional days. Although fading of inflammation paralleled healing of most erosions, a deep necrosis resulting from a large blister at the dorsum of the right foot persisted (Figures 3a–c) so that autologous skin transplantation was performed. Re-examination 11 months after disease onset showed complete clinical remission of disease with re-epithelialization but also scarring of some affected areas (Figures 3d–f).

## Literature Search

Skin manifestations of HSP are characteristic and include a non-thrombocytopenic palpable purpura of the lower extremities and buttocks (1). Rarely, HSP in children may initially present as or evolve into hemorrhagic vesicles and bullae which may result in necrosis of the skin and subsequently scarring (2). To characterize bullous HSP in children we performed a literature search and searched NCBI PubMed for publications using “bullous purpura” as well as the combination of “bullous” and “purpura.” In addition to our patient we identified 41 pediatric HSP patients with bullous skin lesions (Table S1) (3–32). Included were 23 isolated case reports and 6 case series with between 2 and 6 patients. We excluded two of these patients from further analysis since both were later on diagnosed with epidermolysis bullosa and might therefore have dual pathology. We compared the clinical and demographic characteristics of our and the remaining 39 patients (in total 40 patients) with those reported in 4 separate cohorts of unselected pediatric HSP patients using unpaired *t*-test for continuous variables and Fisher‘s exact test for categorial variables (Table 1) (33–36). These cohorts represent the complete clinical range of HSP, however, due to their low prevalence, patients with bullous skin lesions do not significantly contribute to the overall characteristics of these cohorts.

**Table 1:** Comparison of reported patients with bullous Henoch-Schönlein purpura with unselected cohorts.

	**Reported bullous HSP cases (*n* = 40)**	**Saulsbury et al. (33) (*n* = 100)**	**Trapani et al. (34) (*n* = 150)**	**Calvino et al. (35) (*n* = 78)**	**Sano et al. (36) (*n* = 134)**
	**Number (%)**	**Number (%)**	**Number (%)**	**Number (%)**	**Number (%)**
Female	19 (46.3)	43 (43.0)	55 (36.6)	42 (53.8)	63 (47.0)
Age (years)	8.8 ± 3.4	5.9 ± 2.9^*^	6.1 ± 2.7^*^	6.2 ± 3.1^*^	6.3 ± 2.4^*^
Bullous lesions	40 (100)	2 (2.0)	Not reported	1 (1.3)	Not reported
Joint involvement	29 (70.5)	82 (82.0)	111 (74.0)	61 (78.2)	99 (73.9)
Abdominal involvement	23 (57.5)	63 (63.0)	77 (51.3)	57 (73.1)	96 (71.6)
Renal involvement	15 (37.5)	40 (40.0)	81 (54.0)	42 (53.8)	65 (48.5)
Corticosteroid use	26 (65.0)	57 (57.0)	19 (12.6)	18 (23.1)^**^	25 (18.7)^**^

Variables in each cohort were compared to those in reported cases with bullous HSP(

* *p < 0.01*,

** *p < 0.001)*.

The mean age of patients with bullous HSP patients at disease onset was significantly higher compared with each of the four cohorts of unselected HSP patients (Table 1). The prevalence of joint, abdominal and renal involvement in bullous HSP patients was comparable to that of the unselected HSP patient cohorts (Table 1).

In all except two cohorts, bullous HSP patients were treated more often with systemic corticosteroids than the average group of HSP patients (Table 1). In addition, two (5.0%) out of the 40 bullous HSP patients were treated with colchicine, 3 (7.5%) with azathioprine and one (2.5%) with dapsone (Table S1). Three (7.5%) patients were treated with topical steroids and 11 (27.5%) did not receive any anti-inflammatory medical treatment (Table S1).

Bullous lesions resolved in all 11 reported patients who did not receive anti-inflammatory medical treatment and lesions did not re-occur. Hyperpigmentation and/or scar formation has been reported in 11 patients, in 10 of these despite medical treatment. However, detailed information regarding skin sequelae including explicit exclusion of scarring and/or hyperpigmentation is missing in many of the reported patients (Table S1). Interestingly, in 3 out of the 26 (11.5%) corticosteroid treated bullous HSP patients, corticosteroid treatment was already initiated up to 10 days before appearance of hemorrhagic-bullous lesions, mainly due to abdominal involvement (Table S1).

## Discussion

Palpable purpura mainly affecting the buttocks and legs is the characteristic skin manifestations of HSP. The differential diagnosis of these lesions include congenital (e.g., thrombocytopenia, hemophilia) or acquired (purpura fulminans, disseminated intravascular coagulation) forms of bleeding diathesis and other types of vasculitis (e.g., granulomatosis with polyangiitis, eosinophilic granulomatosis with polyangiitis). However, HSP may initially present as or evolve into hemorrhagic vesicles and bullae progressing to cutaneous necrosis which may lead to a diagnostic dilemma (2). The differential diagnosis of blistering skin lesions includes infections (e.g., HSV), toxins (e.g., staphylococcal scalded skin syndrome), autoimmune diseases (e.g., pemphigus), genetic diseases (e.g., epidermolysis bullosa) as well as physical pressure or trauma (37).

Whereas, hemorrhagic-bullous lesions are rather common in adult HSP patients, children with HSP less often develop this type of lesions (< 2%) (38). Due to the wide differential diagnosis, a skin biopsy was performed in our presented case as it has been done in more than half of the reported patients with bullous HSP (Table S1). Histological analysis revealed signs of leucocytoclastic vasculitis in all reported patients, however, IgA deposition could not be detected in all patients (Table S1). This might be explained by the time of biopsy, since deposition of IgA is more difficult to detect in older lesions. Therefore, the biopsy should be obtained from the border of a fresh non-bullous and non-necrotized lesion where proteolytic degradation of IgA is less advanced. Skin biopsy in our patient was performed 10 days after onset of the first bullous lesion but targeted a lesion that evolved <2 days before biopsy. Histological analysis of this skin biopsy revealed the characteristic signs of leucocytoclastic vasculitis with IgA deposition thereby confirming our tentative diagnosis of HSP in or patient.

It has been speculated whether appearance of bullous lesions reflects a more severe disease course or even a distinct disease entity of HSP. The long-term prognosis in HSP is mainly determined by the extent of renal involvement (39). However, our literature review on bullous lesions in pediatric HSP revealed rather less renal involvement in this patient group and seems to contradict this assumption. In most of the published patients the bullous lesions resolved within a few weeks and did not re-occur which led to the assumption of a rather benign and self-limiting course of bullous HSP (2). However, remaining hyperpigmentation and/or scarring have been reported in at least 25% of the reported patients (14, 17, 20, 21, 27, 32). Since information regarding skin sequelae in the disease course of the reported patients is scarce and scarring has not explicitly been excluded in many of the reported patients, the extent of reported skin sequelae rather might be underestimated. In contrast, re-occurrence of active disease has only been described in very few individual cases of bullous HSP and most patients followed a monophasic disease course. However, two patients described to have clinical findings compatible with bullous HSP suffered from re-occurrence of bullous skin lesions 1 and 2.5 years after onset of the first symptoms (16, 18) and were later diagnosed with dominant dystrophic epidermolysis bullosa (DDEB) and recessive-dystrophic epidermolysis bullosa (RDEB), respectively. Histological analysis of a skin biopsy performed at first onset of bullous lesions in the patient with DDEB revealed a non-inflammatory subepidermal bulla in addition to the characteristic signs of leukocytoclastic vasculitis.

The pathophysiology of blistering skin lesions in HSP still remains unclear. Matrix metalloproteinase (MMP)-2 and MMP-9 have been detected in blister fluid and suggested to cause proteolysis of collagen (6). However, the triggers that drive the secretion of these enzymes are not well-understood. Therefore, future research should address the inflammatory mediators that are involved in blister formation in HSP and might be targets of more specific therapies. It might be speculated that bullous skin lesions preferentially develop in HSP patients with intrinsic (e.g., genetic predisposition, general skin fragility) or extrinsic (e.g., physical skin pressure) risk factors but that the appearance of bullous skin lesions does not mirror a distinct pathophysiology of HSP itself.

The optimal treatment regime of patients with bullous HSP is still controversial. The use of systemic corticosteroids in HSP seems to shorten the abdominal pain and arthralgia and decrease the prevalence of purpura (40, 41). Although corticosteroids do not seem to hinder renal manifestation during the disease course, they may reduce the risk of end-stage renal disease (42, 43). There is no clear evidence for a beneficial use of systemic corticosteroids in treating non-bullous skin lesions in HSP. Some authors of published cases with bullous HSP suggested that early systemic treatment with corticosteroids may be beneficial in bullous HSP by promoting faster resolution of skin symptoms and reducing scar formation (17). In contrast, the authors of another systematic case review of patients with bullous HSP concluded that patients with bullous HSP managed expectantly and those managed with systemic steroids display a similar disease course (2).

Whereas, resolution and absence of re-occurrence of lesions has mainly been used as parameters to describe the diseases course and response to therapy in most of the reported patients, less attention has been paid to the potential sequelae of necrotic skin lesions (scarring, hyperpigmentation). Of note, at least 25% of the reported patients with bullous HSP developed some kind of sequelae ranging from hyperpigmented skin areas to deep necrosis necessitating autologous skin transplantation as in our patient (Table S1).

Despite missing of strong evidence due to lack of randomized-controlled trials in bullous HSP, it might be speculated that corticosteroid treatment initiated very early in the disease course of bullous HSP may reduce the severity of necrosis and thereby the extent of skin sequelae. In contrast to this assumption, evolution of bullous skin lesions in HSP patients already receiving high-dose systemic steroids due to abdominal involvement has been documented in 3 cases (7, 21, 22). Additionally, 12 published patients with bullous HSP who showed resolution of skin lesions did not receive any anti-inflammatory medical treatment (3, 4, 6, 12, 14, 15, 18, 20, 28, 30). Although some of these did not develop scarring and/or hyperpigmentation, explicit information about exclusion of skin sequelae is missing in many of these patients. Of note, side effects of systemic corticosteroid treatment have not been described in any of the reported patients, which in our opinion additionally favors the attempt of ameliorating the disease course by using systemic corticosteroids early in the diseases course in bullous HSP.

Steroid-sparing medication has been introduced in patients with long-lasting disease course or inadequate responsiveness to corticosteroids. These treatment regimens included colchicine, azathioprine, or dapsone and have been described in only a few cases (13, 20, 26, 27, 29). Despite the reported benefits in these patients during treatment, the small number of published case does not allow any general conclusion.

## Summary and Concluding Remarks

- Hemorrhagic bullous skin lesions may rarely occur in childhood HSP
- A diagnostic skin biopsy is helpful in the differential work-up of these patients
- Blistering skin lesions in HSP do not generally seem to be associated with more severe extracutaneous/systemic involvement and might not have any prognostic value
- Most of the bullous skin lesions resolve within a few weeks but may lead to necrosis resulting in scarring and/or hyperpigmentation; re-occurrence of bullous lesions may hint at an underlying disease
- The early use of systemic corticosteroids has been suggested in bullous HSP patients but their beneficial effect on the extent of lesions and/or the sequelae of disease has not been analyzed in randomized controlled trials yet
- Supportive care (e.g., avoiding skin pressure, pain control, antibiotic prophylaxis/treatment) is beneficial for all patients

## Ethics Statement

The authors state that written informed consent was obtained from the parents of the patient for the publication of this case report.

## Author Contributions

HM coordinated the writing group. MN and HM performed the literature review. All authors were involved in the patient care, critically reviewed the manuscript and approved the final version.

### Conflict of Interest Statement

The authors declare that the research was conducted in the absence of any commercial or financial relationships that could be construed as a potential conflict of interest. The reviewer KM and handling Editor declared their shared affiliation.

## Abbreviations

DDEB: Dominant dystrophic epidermolysis bullosa
(HSP): Henoch-Schönlein Purpura
HSV: Herpes simplex virus
MMP: matrix metalloproteinase
RDEB: recessive dystrophic epidermolysis bullosa.
