# Supplementary material for: Hemorrhagic Bullous Henoch-Schönlein Purpura: Case Report and Review of the Literature
Source: Front Pediatr. 2019 Jan 22;6:413. doi: 10.3389/fped.2018.00413 (PMC6349767; doi:10.3389/fped.2018.00413)
Supplement: Supplementary file 1 [file Data_Sheet_1.PDF]

Table S1 – Summary of 43 reported patients with bullous Henoch-Schoenlein Purpura

No.	Age	Sex	Localization of bullous lesions	Size of lesions	Organ involvement Joints	Abdomen	Renal	Biopsy: IgA-deposition	Medical Treatment	Re-occurence of lesions	Skin sequelae	Documented Follow-up	Reference
1	5	m	legs, arms	unknown	yes	yes	yes	not performed	no treatment	no	unknown	unknown	Garland *et al*
2	7	f	legs	unknown	yes	no	no	not performed	no treatment	no	unknown	unknown	Bari *et al*
3	5	m	pinnas, gum, hands, legs	2-50 mm	yes	yes	yes	no	PS	no	none	unknown	Wananukul *et al*
4	10	m	legs	not reported	yes	yes	no	no	no treatment	no	unknown	12 months	Kobayashi *et al*
5	7	f	feet, ankles	not reported	yes	yes	no	yes	PS	no	none	12 months	Saulsbury *et al*
6	3	f	legs, feet	5-20 mm	yes	yes	yes	not performed	PS, started 10 days before onset of bullae	no	none	6 months	
7	7	f	legs	unknown	yes	no	no	no	HC	no	none	unknown	Liu *et al*
8	6	m	legs	unknown	yes	yes	no	no	HC	no	pigmentation	unknown	
9	4	m	face, hands, buttocks, legs	5-20mm	yes	yes	no	yes	PS, MPS	no	unknown	3 months	Ishii *et al*
10	8	m	ankles, feet	- 35 mm	yes	yes	no	not performed	no treatment	no	none	6 months	Leung *et al*
11	14	m	feet	not reported	no	yes	no	no	naproxen, colchicine	Similar lesions 2 years earlier	none	3 months	Chan *et al*
12	4	f	legs	not reported	yes	no	no	not performed	no treatment	no	none	3 months	Aydinoz *et al*
13	10	m	hands, legs, feet	not reported	yes	yes	yes	yes	PS	no	unknown	3 months	Abdul-Ghaffer *et al*
14	9	f	Arms, legs, feet	3-20 mm	no	yes	no	yes	PS	no	scarring	12 months	Junior *et al*
15	9	f	feet	5-10 mm	yes	no	yes	not performed	PS	no	none	14 months	
16	6	f	feet	10-15 mm	yes	no	no	yes	no treatment	no	pigmentation	10 months	

No.	Age	Sex	Localization of bullous lesions	Size of lesions	Organ involvement			Biopsy: IgA-deposition	Medical Treatment	Re-occurence of lesions	Skin sequelae	Documented Follow-up	Reference
					Joints	Abdomen	Renal						
17	8	m	hands, feet	not reported	yes	yes	no	yes	PS	re-occurence after 2.5 years [1]	unknown	2.5 years	Maguiness *et al*
18	15	f	legs	not reported	no	no	no	no	topical corticosteroids	no	none	not reported	
19	8	m	trunks, legs	not reported	yes	yes	no	yes	topical corticosteroids	not reported	unknown	not reported	
20	8	m	legs	not reported	no	no	no	not performed	no treatment	not reported	unknown	not reported	
21	11	f	legs	not reported	no	yes	no	yes	no treatment	not reported	unknown	not reported	
22	10	f	legs	not reported	no	yes	no	not performed	MPS	not reported	unknown	not reported	
23	6	m	arms, legs	not reported	no	yes	yes	yes	PS	no	scarring, Pigmentation	22 months	den Boer *et al*
24	10	m	legs	not reported	no	no	yes	no	PS	no	mild scarring	17 months	
25	16	f	legs	not reported	yes	yes	no	yes	PS	re-occurence after 1 year [1]	scarring	12 months	Abdulla *et al*
26	9	f	arms, legs	2-30 mm	yes	yes	yes	not performed	MPS, PS	no	scarring, pigmentation	12 months	Trapani *et al*
27	11	m	ankles, legs	not reported	yes	no	no	not performed	no treatment	no	unknown	28 months	
28	7	f	face, arms, trunks, legs	not reported	yes	no	yes	not performed	MPS, PS, AZA	no	unknown	5 months	
29	3	f	ankles, feet	not reported	yes	yes	yes	no	PS, started 3 days before onset of bullae	no	mild scarring	6 months	Park *et al*
30	9	f	ankles, feet	not reported	yes	no	yes	not performed	PS	no	unknown	not reported	Raymond *et al*
31	8	f	arms, legs	15 mm	no	yes	no	not performed	PS, started > 3 days before onset of bullae	no	none	not reported	Hömber *et al*
32	6	m	legs, feet	20-30 mm	yes	yes	no	not performed	PS	no	none	12 months	Matsubara *et al*
33	14	m	feet	not reported	no	yes	yes	not performed	PS	no	unknown	4 weeks	Parikh *et al*

No.	Age	Sex	Localization of bullous lesions	Size of lesions	Organ involvement			Biopsy: IgA-deposition	Medical Treatment	Resolution of lesions	Skin sequelae	Documented Follow-up	Reference
					Joints	Abdomen	Renal						
34	4	f	ankles, legs	not reported	yes	yes	yes	not reported	PS	no	unknown	4 months	Kocaoglu *et al*
35	9	f	trunk, legs	3-50 mm	no	yes	yes	yes	DEX, PS, AZA	no	unknown	5 years	Mehra *et al*
36	3	m	arms, legs	not reported	no	no	no	not reported	no treatment	no	unknown	6 months	Gratin *et al*
37	14	f	trunk, arms, legs	not reported	yes	no	no	yes	PS, dapsone	no	mild pigmentation	6 months	Chen *et al*
38	9	m	ankles, feet	not reported	yes	no	no	not performed	topical corticosteroids	no	unknown	1 month	Hopper *et al*
39	5	m	legs, face	not reported	yes	yes	no	yes	PS, colchicine	re-occurunce w/o colchicine	unknown	3 years	Allali *et al*
40	14	f	legs	not reported	yes	no	no	yes	HC, PS, AZA	no	unknown	2 months	Hasbun *et al*
41	15	m	legs, ankles	2-20 mm	yes	yes	no	no	HC, PS	no	mild scarring	12 months	Su *et al*
42	5	m	legs, ankles, feet	5-40 mm	yes	yes	no	yes	PS	no	scarring, skin transplant	11 months	present report

AZA azathioprine, *DEX* dexamethasone, *HC* hydrocortisone, *MPS* methylprednisolone, *PS* prednisolone
[1] this patient was diagnosed with recessive-dystrophic epidermolysis bullosa
[2] this patient was diagnosed with dominant dystrophic epidermolysis bullosa
